# Fabrication of Porous Anorthite Ceramic Insulation Using Solid Wastes

**DOI:** 10.3390/ma17071478

**Published:** 2024-03-24

**Authors:** Mia Omerašević, Vladimir Pavkov, Milena Rosić, Marija Egerić, Snežana Nenadović, Dušan Bučevac, Nebojša Potkonjak

**Affiliations:** Vinča Institute of Nuclear Sciences, National Institute of the Republic of Serbia, University of Belgrade, Mike Petrovica Alasa 12-14, 11000 Belgrade, Serbia; pavkow@vin.bg.ac.rs (V.P.); mrosic@vin.bg.ac.rs (M.R.); egericmarija@vin.bg.ac.rs (M.E.); msneza@vin.bg.ac.rs (S.N.); bucevac@vin.bg.ac.rs (D.B.); npotkonjak@vin.bg.ac.rs (N.P.)

**Keywords:** anorthite, banana peel, waste seashells, porous ceramics, thermo-mechanical properties, microstructure

## Abstract

Porous anorthite (CaAl_2_Si_2_O_8_) ceramics, suitable for thermal insulation in buildings, were obtained using waste seashells as a source of CaO, kaolin as a source of Al_2_O_3_ and SiO_2_ and banana peel as a pore former. Changing the volume of banana peel as well as the processing temperature was found to be an effective approach to control the thermo-mechanical properties of the obtained anorthite ceramics. The sintering of powder compacts containing up to 30 wt% banana peel at temperatures ranging from 1100 to 1200 °C resulted in anorthite ceramics possessing up to 45% open porosity, a compressive strength between 13 and 92 MPa, a bulk density between 1.87 and 2.62 g/cm^3^ and thermal conductivity between 0.097 and 3.5 W/mK. It was shown that waste materials such as seashells and banana peel can be used to obtain cost-effective thermal insulation in buildings.

## 1. Introduction

Heating and cooling in buildings contribute to an impressive 40% of the world’s primary energy consumption and are responsible for 33% of annual CO_2_ emissions [1]. It is also interesting to note that the greenhouse gas emissions resulting from the energy consumption for heating and cooling in buildings significantly exceed those from traffic and industrial activities [2]. At present, the use of thermal insulation materials in buildings is of great importance due to their contribution to climate change mitigation. There is a global tendency towards reducing the consumption of energy required for heating and cooling in buildings. Therefore, thermal insulation technology is becoming important in preventing both the gain and the loss of heat through the building envelope. Currently, polystyrene and polyurethane are the most commonly used insulation materials for buildings. Unfortunately, these materials have some drawbacks. Although polyurethane foams are normally harmless when in their solid form, the process of foam spray deposition is quite harmful due to the evaporation of toxic gasses such as isocyanates. On the other hand, the main disadvantage of polystyrene, which is mostly used in the form of an insulation board, lies in the fact that the boards glued to the facade can transfer fires to higher floors [3].

Porous ceramics were found to be a promising material for internal wall lining owing to their good combination of properties such as low density, high strength, low thermal conductivity and excellent fire resistance. Firing (sintering) is one of the most frequently used techniques for porous ceramics’ fabrication [4,5]. Similar to ceramic tiles’ firing, sintering can be employed to make rigid, porous ceramic panels, starting with ceramic preforms which can be obtained through different methods such as powder pressing, slip casting, or tape casting. Anorthite ceramics are especially convenient for building insulation owing to their low theoretical density of only 2.76 g/cm^3^ and their relatively low production cost. Anorthite is calcium aluminosilicate (CaAl_2_Si_2_O_8_), which can be obtained using commercial powders of alumina, silica and oxides of calcium. Anorthite can be also obtained by sintering a Ca-LTA zeolite at temperatures ranging from 1050 to 1400 °C [6]. The use of commercial powders is justified only if a high degree of purity of the material is required. In this case, for insulation purposes, low-cost sources of these oxides are preferred. The most common inexpensive sources used that obtain anorthite are kaolin and CaCO_3_, in the form of calcite. Gypsum, marble powder, quartz and Ca(OH)_2_ have also been used. Kaolin is very often used in the fabrication of alumosilicate ceramics as it is relatively inexpensive and an abundant source of silicon and aluminum. The cost of anorthite’s fabrication can be further lowered by using waste (side products), such as empty seashells, as a source of calcium. The fishery industry [7] produces a large amount of empty seashells which normally end up in a landfill. Odors and the breeding of flies and mosquitoes are normally widespread during the natural decomposition of these untreated seashells. In some cases, toxic compounds that could harmfully affect the living environment are also produced [8]. It is known that seashell waste consists of marine organisms’ exoskeletons, which contain around 90–99 wt% CaCO_3_, which makes seashells an appropriate replacement for conventional sources of CaCO_3_ as well as CaO [9].

In order to eliminate this waste by using it as a source of CaCO_3_, some researchers have been trying to use seashells in the construction industry. It has been shown that shells can be used not only as a filler [10] but also as a partial replacement for a coarse aggregate [11], sand [12], bricks [13] and cement [14]. Another waste that creates an environmental and disposal problem is banana peel. Banana is one of the most consumed fruits in the world, and the annual production of banana peel waste is almost 36 million tons [15]. The peels from bananas are frequently discarded into the garbage, as well as environment, without any preparation. Consequently, there is a constant need for the removal of this waste from the environment and its utilization instead [16]. Banana peel can be used in biofuels and fertilizer production [15], in the food industry, as a biomaterial for the adsorption of heavy metals in waste streams [17,18], etc. In this work, banana peel, as an organic material, will be used as a pore former in the fabrication of porous anorthite. Therefore, in terms of waste remediation as well as thermal insulation in buildings, porous anorthite ceramics could be a promising material that offers environmental acceptability and sustainability. In general, the ceramics industry has a promising capacity for the utilization of different kinds of waste. It is well known that aluminum plant waste and anodizing sludge have been used for the manufacturing of insulation bricks [19] and environmentally clean composites [20]. Furthermore, fly ash has been used for the production of mullite ceramic foams [21], whereas red mud has been used to obtain ceramic floor tiles [22], etc. A high processing temperature allows for different treatments of waste including their burnout, decomposition, or even transformation into useful compounds.

This work aims to evaluate the feasibility of using seashell waste as a source of CaO, kaolin as a source of Al_2_O_3_ and SiO_2_ and banana peel as a pore former in the manufacturing of sustainable ceramics for thermal insulation. As per a survey of the literature, no research reports have been found on the production of anorthite-based porous ceramics using this combination of waste as starting materials. The open porosity, microstructure, thermal conductivity at room temperature, phase composition and compressive strength of our samples were systematically investigated. 

## 2. Materials and Methods

### 2.1. Materials

Seashell waste, from the Aegean Sea coast, was used as a source of CaCO_3_ (calcium carbonate). To remove sand particles and other soluble impurities the seashells were washed with warm distilled water and dried at 50 °C. In order to obtain a particle size smaller than 0.2 mm, the shells were milled in a tungsten carbide vibrating cup mill, Fritsch Pulverisette 9, Idar-Oberstein, Germany. The grinding lasted for 45 min, in a dry state, at 800 rpm.

Banana peels obtained from a local market were cut into small pieces, dried for 24 h at 150 °C and subsequently ground into powder using a laboratory ball mill consisting of HDPE bottles filled with alumina balls and placed on roller machine, MTI Corporation, Richmond, CA, USA. H.F. Hassan et al. [23] conducted a comprehensive analysis of banana peels’ composition. When it comes to organic compounds, it was found that banana peel contains proteins (1.95%), fat (~6%) and carbohydrates (~12%). There are also inorganic compounds present containing elements such as Na (115.1 mg/100 g), P (211.3 mg/g), Ca (59.1 mg/g), Fe (47 mg/g), Mg (44.5 mg/g) and K (4.39mg/g), as well as Zn, Cu, Mn < 1 mg/g. The obtained powder was sieved to a particle size fraction < 0.5 mm [18]. Raw kaolin from Rudovci (Lazarevac, Serbia) was calcined at 750 °C for 3 h with a constant heating rate of 5 °C/min in order to obtain metakaolin. The physicochemical characterization of kaolin has been presented in previous research [24]. 

### 2.2. Methods

**Figure 1:**
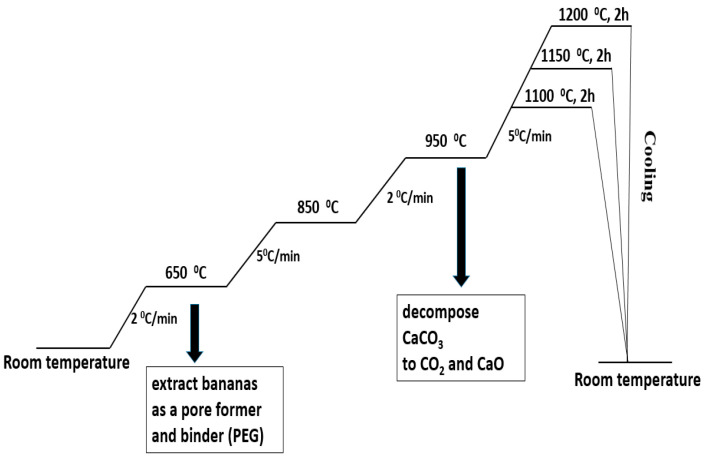
The heating regime of the thermal treatment of a mixture of seashell, metakaolin (MK-SH) and banana peel.

Metakaolin (MK) and seashells (in a stoichiometric ratio for obtaining anorthite) were mixed for 12 h in a plastic bottle (Nalgene Wide-Mouth HDPE IP2, 500 mL, Nalgene, Rochester, NY, USA)) using Al_2_O_3_ balls as a milling media and distilled water as a liquid vehicle. The obtained powder composite of the MK and seashells was mixed for 12 h with different amounts of banana peel as the pore former and the presence of 5 wt% polyethylene glycol (PEG) as a binder. The obtained powders were dried and sieved. To make green compacts with a height of ~12 mm, the powders were uniaxially pressed. The applied pressure was 50 MPa and the mold diameter was 8 mm. Powder compacts containing 0, 10, 20 and 30 wt% banana peel were heat-treated in air at 1100 °C, 1150 °C and 1200 °C for 2 h in order to determine the optimum sintering temperature. The heating regime is presented in Figure 1. The samples were slowly heated, at a rate of 2 °C/min, to 650 °C in order to remove organic components, i.e., PEG and banana peel. The heating rate was increased by 5 °C/min to the temperature range 650–850 °C and subsequently lowered by 2 °C/min to the temperature range 850–950 °C in order to allow the slow release of the gasses created during the decomposition CaCO_3_ to CO_2_ and CaO. Eventually, the samples were heated to their final sintering temperatures at a heating rate of 5 °C/min. After sintering, the samples were furnace-cooled to room temperature. 

An XRPD (X-ray powder diffraction) analytical technique was performed using a Rigaku diffractometer (Ultima IV, Tokyo, Japan). The collection range (2*θ*) of the diffraction data was from 5° to 70° with filtered Cu Kα radiation, a scan speed of 5°/min and a step size of 0.02°. PDXL2 software (version 2.0.3.0) was used for the identification of the phases present. The standard ASTM C373-14a method [25] was used to determine the bulk density and open porosity of six samples of each composition. The microstructure analysis of anorthite-based ceramics was conducted using a MIRA3 TESCAN (Brno, Czech Republic) scanning electron microscope (SEM). The ceramic samples were coated with a 15 nm layer of gold before analysis to provide electrical conductivity to the surface. The applied magnification was 5000× for all samples. Their compressive strength was examined using a universal testing machine, model 1185, manufactured by Instron (Norwood, MA, USA), at a crosshead speed of 1 mm/min. The mean value of six samples of each composition was calculated. The cylindrical samples were cautiously sanded to make the bottom and upper surfaces parallel. The hot-disk method was utilized to determine the thermal conductivity of the samples. A hot-disk thermal analyzer (TPS3500, Hot disk AB Co., Göteborg, Sweden) was used to test five samples of each composition at 30 °C. ISO standard No.22007-2:2022 [26] was applied as the standard to measure the thermal properties of the ceramics using the hot-disk method.

## 3. Results and Discussion

### 3.1. Phase Composition

**Figure 2:**
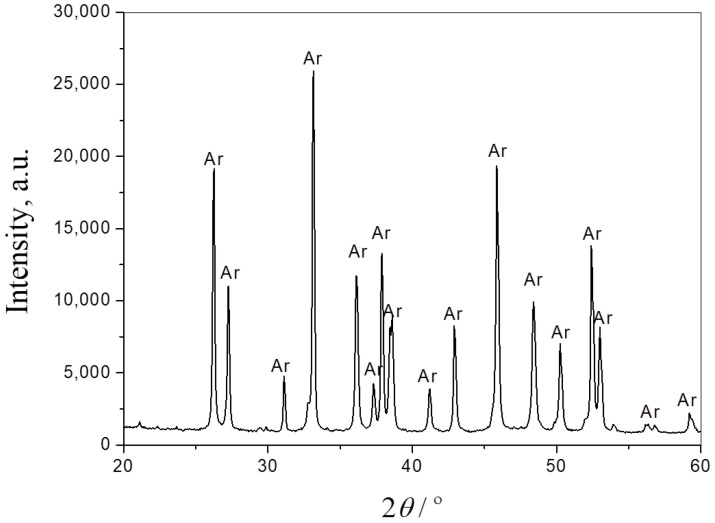
XRPD pattern of seashell powder. Ar—aragonite.

The phase composition of the seashells, starting composite powder and the sintered samples was obtained by XRPD analysis. Figure 2 presents the XRPD pattern of seashell powder. As can be seen from the diffractogram, the only phase in this material is CaCO_3_, in the form of aragonite (JCPDS Card No. 41-1475). It is one of the three most common naturally occurring crystal forms of calcium carbonate. 

**Figure 3:**
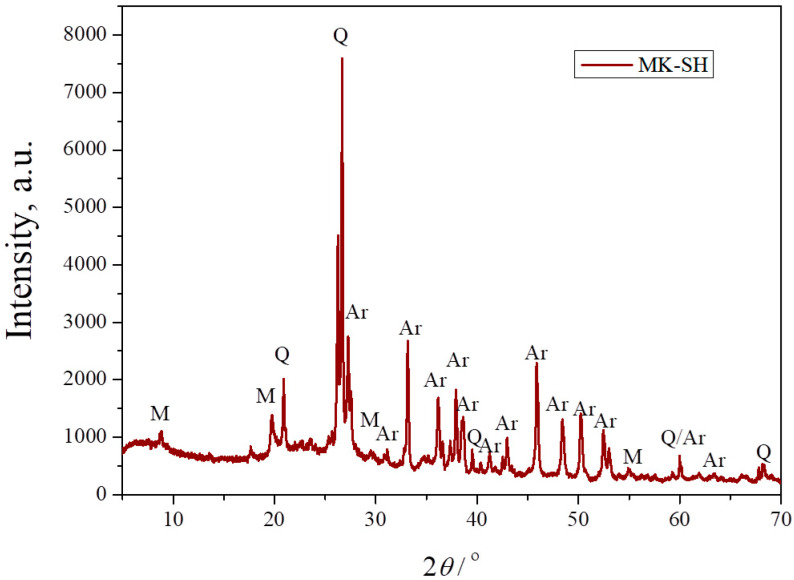
XRPD pattern of a mixture of metakaolin and seashells (MK–SH) with anorthite stoichiometry: Q—quartz, M—muscovite, Ar—aragonite.

Figure 3 shows the XRPD pattern of the composite powder consisting of calcined kaolin and seashells in a stoichiometric ratio for the production of an anorthite phase. As mentioned, kaolin is an aluminosilicate clay mainly consisting of the clay mineral kaolinite, accompanied by one or more crystalline phases. After calcination at 750 °C, kaolin transforms into metakaolin, which is a predominantly anhydrous form of kaolinite. As Figure 3 evidences, the strongest characteristic peaks of kaolinite, located at ~12° and ~25°, were not detected, confirming the complete transformation of kaolin into metakaolin at 750 °C. The XRPD pattern also shows the presence of three crystalline phases. The first one is the carbonate mineral aragonite, which originates from the seashells, as confirmed in Figure 2. The other two crystalline phases, namely quartz and muscovite, could be identified as components of the starting kaolin [24]. The appearance of broad bands located on the far left of the XRPD pattern and between 18° and 25° indicates the presence of amorphous structures. The presence of an amorphous phase is expected, knowing that the dehydration of kaolinite causes the breakage of its ordered structure.

**Figure 4:**
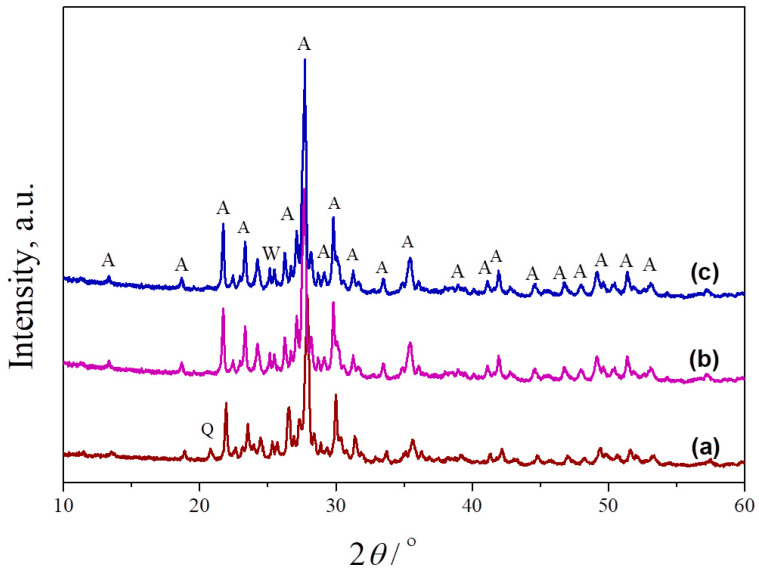
XRPD patterns of crushed samples of powder compacts containing no banana peel, obtained after 2 h long sintering at different temperatures: (**a**) 1100 °C, (**b**) 1150 °C and (**c**) 1200 °C. A—anorthite, Q—quartz, W—wollastonite.

Mixtures of metakaolin and seashell, containing different amounts of banana peel as a pore former, were sintered at 1100 °C, 1150 °C and 1200 °C to obtain porous anorthite-based ceramics. Figure 4 presents the phase composition of samples sintered at different temperatures. As can be seen, an anorthite phase was formed at 1000 °C (indexed by JCPDS Card No.: 01-089-1472). The intensities of the characteristic anorthite peaks are their highest at 1200 °C. The peak located at 25.4° in the XRPD pattern of the sample heated to 1100 °C indicates the presence of a small amount of a wollastonite phase (indexed by JCPDS Card No.: 01-075-1396), which remained even at 1200 °C. The presence of wollastonite (CaSiO_3_) is the result of an Al (Al_2_O_3_) deficiency in the starting kaolin. Unlike wollastonite, the presence of a small amount of quartz was confirmed only in the sample sintered at the lowest temperature of 1100 °C due to an incomplete reaction between the starting oxides. The main characteristic peak of quartz (indexed by JCPDS Card No.: 01-070-2517) is located at 21°.

### 3.2. Bulk Density and Open Porosity

**Figure 5:**
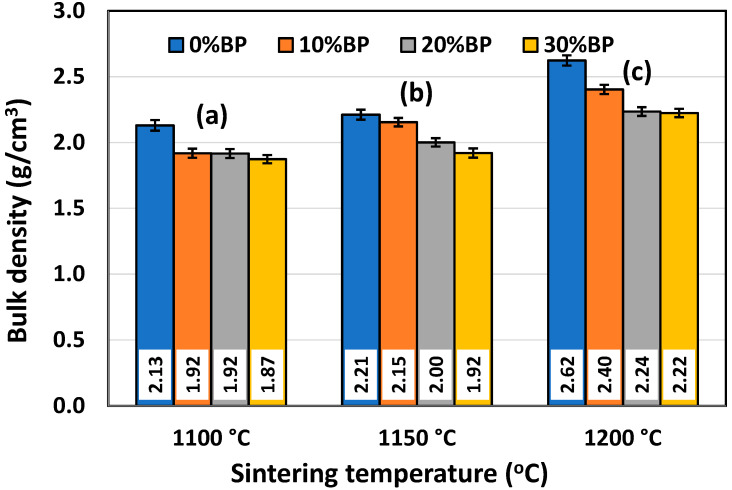
Bulk density of anorthite ceramics containing different amounts of banana peel (BP), obtained after 2 h long sintering at different temperatures: (**a**) 1100 °C, (**b**) 1150 °C and (**c**) 1200 °C. Theoretical density (TD) of anorthite is 2.76 g/cm^3^.

**Figure 6:**
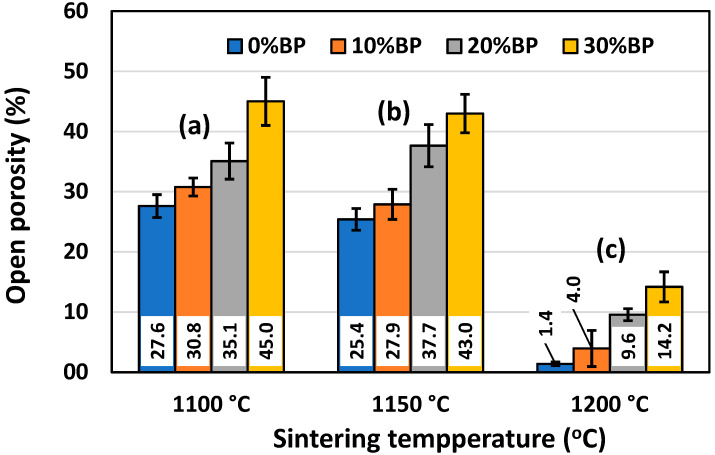
Open porosity of anorthite ceramics containing different amounts of banana peel (BP), obtained after 2 h long sintering at different temperatures: (**a**) 1100 °C, (**b**) 1150 °C and (**c**) 1200 °C.

It is well known that density and porosity are two very important properties for insulating ceramics as they strongly affect their thermal conductivity. The mean values of the bulk density (BD) and open porosity (OP) of samples containing different amounts of banana peel and sintered at different temperatures are presented in Figure 5 and Figure 6, respectively. It can be observed in Figure 5 that the bulk density of the samples containing different amounts of banana peel generally increased with the sintering temperature. The highest density values measured in samples sintered at 1200 °C are the result of the fast densification that takes place at high temperatures. When it comes to the samples containing different amounts of banana peel that were sintered at 1200 °C, it can be seen that the highest density of 2.62 g/cm^3^ was measured in the samples without banana peel, whereas the lowest density of 2.22 g/cm^3^ was measured in samples containing 30 wt% banana peel. This is expected, knowing that organic compounds such as banana peel burn out at 600–700 °C, creating additional porosity which cannot be completely removed during the sintering process. This trend was also observed for lower sintering temperatures. As can be seen in Figure 5, the sample containing 30 wt% banana peel, sintered at 1100 °C, had the lowest bulk density of all the samples, only 1.87 g/cm^3^, which was the result of both slow densification and a large amount of pore former, i.e., banana peel.

Similar to Figure 5, Figure 6 shows the effect of the sintering temperature and amount of banana peel on the open porosity of sintered anorthite samples. It can be seen that the open porosity of samples containing different amounts of banana peel generally decreased with the sintering temperature. The sample sintered at 1200 °C without banana peel as a pore former had the lowest value of open porosity, only 1.40%. On the other hand, the samples containing 30 wt% banana peel and sintered at 1100 °C for 2 h had the highest open porosity of all the samples, reaching 45.01%. A relatively large drop in open porosity was measured in samples with different amounts of banana peel sintered at 1200 °C for 2 h, as a result of the accelerated sintering process (densification) at high temperatures. A relatively large porosity of 45% was achieved owing to the two processes that take place simultaneously during the sintering of samples containing 30% banana peel at 1100 °C. One process is the burnout of banana peel, whereas the other one is the decomposition of seashells (CaCO_3_) into CaO and CO_2_ gas at ~900 °C. It is evident that careful control of the sintering temperature and the amount of banana peel used is an efficient way to obtain materials with porosities varying from 1.40% to 45.01%. The rate of the sintering process can also be described by measuring sample shrinkage after sintering, since pore elimination (densification) is inevitably followed by sample shrinkage.

### 3.3. Shrinkage

**Figure 7:**
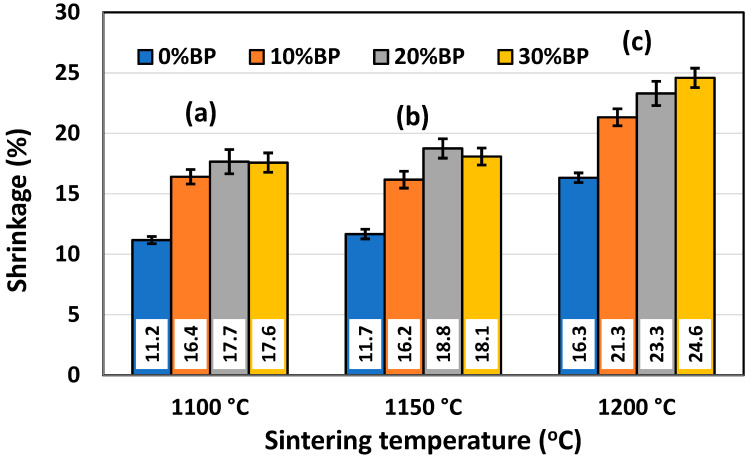
Shrinkage of anorthite samples containing different amounts of banana peel (BP), obtained after 2 h long sintering at different temperatures: (**a**) 1100 °C, (**b**) 1150 °C and (**c**) 1200 °C.

The shrinkage of powder compacts consisting of metakaolin, seashells and different amounts of banana peel was measured after sintering. Figure 7 shows the effect of sintering temperature on the shrinkage of samples containing different amounts of banana peel. Obviously, the increase in the sintering temperature from 1100 °C to 1200 °C led to a shrinkage increase due to the faster sintering process occurring at a high temperature. It is important to point out that the shrinkage values for samples sintered at 1100 °C and 1150 °C are quite similar, ranging from ~11.17% in samples without banana peel sintered at 1100 °C to 18.75% in samples containing 30 wt% banana peel sintered at 1150 °C. A somewhat larger shrinkage was measured after sintering at 1200 °C. The values ranged from 16.33% in samples without banana peel to 24.59% in samples containing 30 wt% banana peel. The large shrinkage of samples containing 30 wt% banana peel sintered at 1200 °C was the result of both the high sintering temperature and considerable porosity created due to pore former burnout, which takes place at a temperature lower than the sintering temperature. The elimination of this porosity during the sintering process causes a significant consolidation and, therefore, shrinkage of the samples.

### 3.4. Compressive Strength

**Figure 8:**
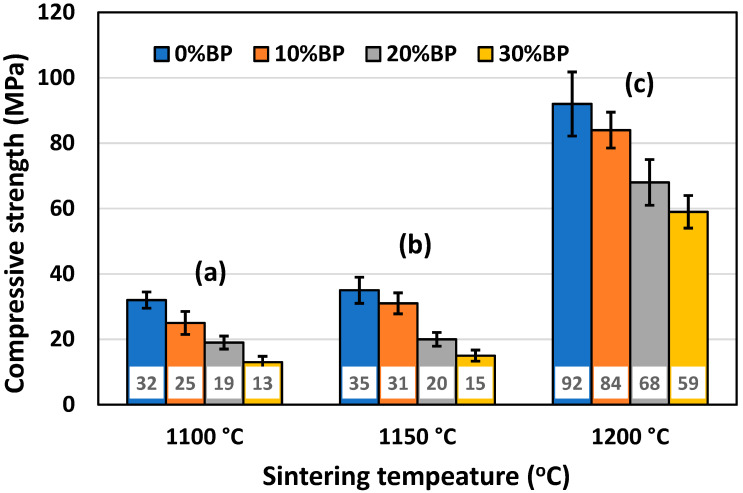
Compressive strength of anorthite ceramics containing different amounts of banana peel (BP), obtained after 2 h long sintering at different temperatures: (**a**) 1100 °C, (**b**) 1150 °C and (**c**) 1200 °C.

Besides porosity, one of the most important properties of ceramic insulation materials is their compressive strength. It is essential for insulation materials to possess a sufficiently high value of compressive strength, which allows for the easy handling and rigidity of the installed material. The mean values of the compressive strength of samples with different amounts of pore former, sintered at different temperatures, are presented in Figure 8. It can be seen that porosity strongly affects compressive strength. The highest compressive strength was measured in samples sintered at 1200 °C, which had the lowest porosity (Figure 6). The compressive strength of samples containing 30 wt% banana peel was found to be 59 MPa, whereas the compressive strength of samples without banana peel reached a value of 92 MPa. Samples sintered at 1100 °C and 1150 °C have similar compressive strength values, but these much lower than those measured for samples sintered at 1200 °C. Samples containing 30 wt% banana peel sintered at 1100 °C and 1150 °C had the lowest values of compressive strength, 16 MPa and 21 MPa, respectively. This finding is expected, knowing that these samples possess high porosities (Figure 6) reaching values of ~45% and ~43%, respectively.

### 3.5. Thermal Conductivity

**Figure 9:**
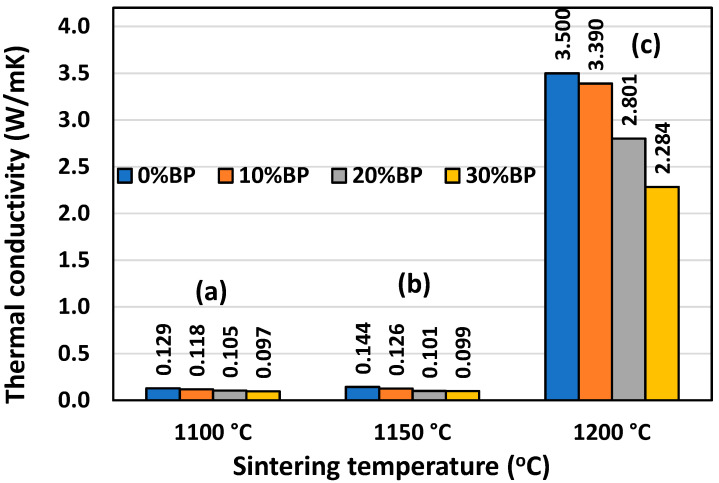
Thermal conductivity of anorthite ceramics containing different amounts of banana peel (BP), obtained after 2 h long sintering at different temperatures: (**a**) 1100 °C, (**b**) 1150 °C and (**c**) 1200 °C. Error bars are not presented (visible) due to very small standard deviation.

Similar to the compressive strength, thermal conductivity is also strongly affected by porosity. Figure 9 shows that the highest conductivity was measured in samples sintered at 1200 °C, which possess the lowest open porosity, ranging from 1.40% to 14.20% (Figure 6). The conductivity of the sample containing 30 wt% banana peel was 2.284 W/mK, whereas the conductivity of samples without banana peel reached a maximum value of 3.50 W/mK. This value was very close to the theoretical value of the thermal conductivity of anorthite, which has been reported to be 3.67 W/mK [27]. Samples with a higher porosity, such as those sintered at 1100 °C and 1150 °C, have much lower conductivity. The thermal conductivities of the samples containing different amounts of banana peel sintered at 1100 °C and 1150 °C are very similar. The lowest thermal conductivity of 0.097 W/mK was measured in samples containing 30 wt% banana peel and sintered at 1100 °C. Such a low conductivity value is considered to be the result of its high open porosity of 45%. The conductivity of the samples without banana peel that were sintered at 1100 °C, which have a porosity of 27.61%, was somewhat higher, reaching a value of 0.129 W/mK.

### 3.6. Microstructure

The properties examined in the previous sections continuously changed with the amount of banana peel, which varied from 0 wt% to 30 wt%. Therefore, our microstructural analysis was focused on the samples without banana peel and samples containing 30 wt% banana peel. 

**Figure 10:**
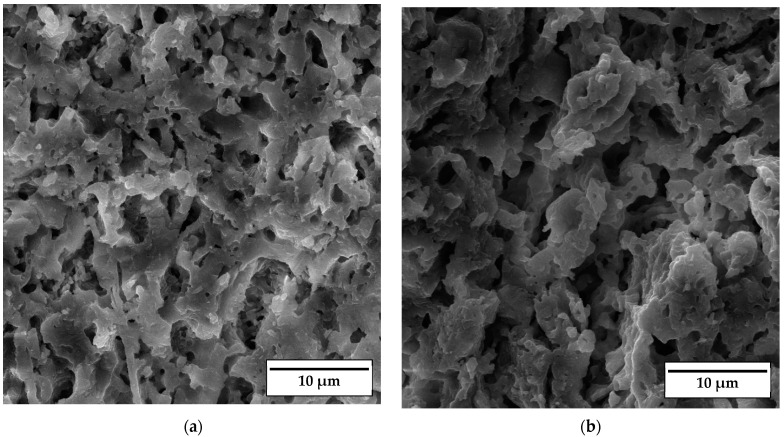
SEM micrographs of fracture surfaces of samples sintered at 1100 °C for 2 h containing (**a**) 0 wt% and (**b**) 30 wt% banana peel.

**Figure 11:**
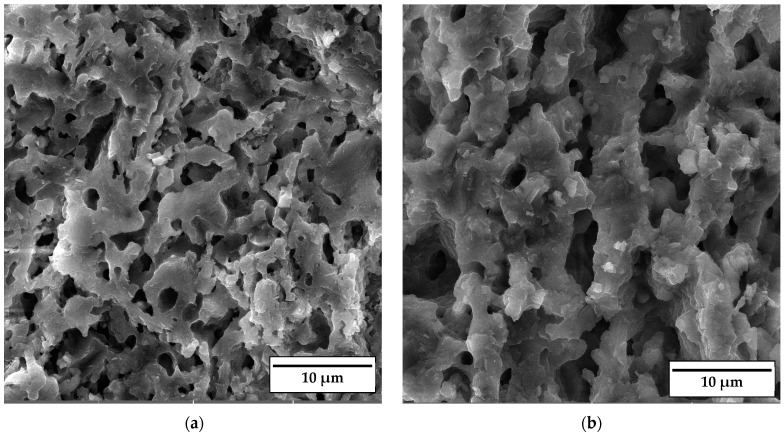
SEM micrographs of fracture surfaces of samples sintered at 1150 °C for 2 h containing (**a**) 0 wt% and (**b**) 30 wt% banana peel.

SEM micrographs of the fracture surface of anorthite samples containing 0 wt% and 30 wt% banana peel and sintered at 1100 °C are presented in Figure 10. Although the microstructures of these two samples are very similar, it is evident that both the porosity and pore size are larger in the sample containing 30 wt% banana peel (Figure 10b). The open porosity of the samples without banana peel was 27.61%, whereas the porosity of the samples containing 30 wt% banana peel was ~45%, as confirmed in Figure 6. It can be concluded that the addition of 30 wt% banana peel increased the open porosity of the ceramics by almost 17%. It is also worth mentioning that a certain amount of porosity in all samples is created due to the decomposition of seashells (CaCO_3_) and the consequential CO_2_ gas release that takes place during sintering [8]. In general, high porosity is a crucial property of insulating materials, as it is normally accompanied by low thermal conductivity. On the other hand, high porosity is typically accompanied by low strength, which is not key property of insulating ceramics and must reach certain level which will allow for easy material handling. Bearing this in mind, one can see in Figure 10 that both samples possess a skeleton/framework which consists of sintered material with small pores of a diameter below ~2 µm. This framework provides the rigidity which is necessary for the application of anorthite as an insulation material. The compressive strength of samples sintered at 1100 °C decreased from 32 MPa in the sample with no banana peel (27.61% porosity) to 13 MPa in the sample with 30% banana peel (~45% porosity) (Figure 6 and Figure 8). It is evident that the variation of the amount of banana peel allows us to adjust the preferred ratio between the porosity and compressive strength of samples sintered at 1100 °C. As discussed earlier, a further increase in the sintering temperature to 1150 °C does not affect properties such as open porosity, compressive strength and thermal conductivity in any significant way. A similar conclusion can be drawn from Figure 11, which shows that the microstructures of the samples containing 0 wt% and 30 wt% banana peel sintered at 1150 °C are very similar to those of the samples with the same amount of banana peel sintered at 1100 °C (Figure 10). A careful comparison of Figure 10b and Figure 11b reveals that the framework in the sample sintered at 1150 °C is less porous than that in the sample sintered at 1100 °C. It appears that the increase in the sintering temperature by 50 °C increases the diffusion coefficient, which accelerates the densification process and therefore the elimination of pores, which causes a small increase in density, from 1.87 g/cm^3^ in samples containing 30 wt% banana peel sintered at 1100 °C to 1.92 g/cm^3^ in samples containing 30 wt% banana peel sintered at 1150 °C (Figure 5). Densification due to the formation of a liquid phase is not expected, knowing that the melting points of anorthite as well as minor phases such as quartz and wollastonite are considerably higher than the sintering temperature. Despite the increase in the densification rate, the open porosity of the sample containing 30 wt% banana peel sintered at 1150 °C was very close to that of the sample sintered at 1100 °C. These are two highly porous materials with open an porosity of 42.98% and ~45%, respectively. This high porosity originates from the large, interconnected pores that are observed at the fracture surfaces presented in Figure 10b and Figure 11b. These pores and interfaces primarily decrease the section area through which heat is transported by phonons [27]. Knowing that thermal conductivity decreases with porosity, it is quite expected that these highly porous samples have the lowest values of thermal conductivity. For example, Lei Han et al. [28] obtained porous platelet-like anorthite ceramics by gel casting, using H_3_BO_3_ and melamine as sintering/crosslinking agents. Although the highly porous ceramics (71% porosity) they obtained at 1000 °C showed good thermal properties, their compressive strength was very low, only 5.7 MPa. Furthermore, S. K. S. Hossain and P. K. Roy [29] used fly ash and seashells as ingredients to fabricate, at 1100 °C, highly porous Ca–aluminosilicate-based ceramics with a thermal conductivity at 30 °C of 0.102 W/mK and a compressive strength of 7.27 MPa. Unlike their thermal conductivity, which was similar to the thermal conductivity of the samples with 30 wt% banana peel obtained in the present study, after sintering at 1150 °C, their compressive strength of 7.27 MPa was two times lower than the compressive strength of the samples created in the present study. Hence, the important benefit of the present study is that the low thermal conductivity of these samples is accompanied by their sufficiently high compressive strength, necessary for easy handling. In addition, Y. Han et al. [30] generated some very interesting results. They obtained, with the help of a hydrous foam–gel casting process, very porous anorthite ceramics of 91% porosity and an ultra-low thermal conductivity of 0.018 W/mK. The main drawback is that this material was so brittle that the authors were not able to measure its compressive strength.

**Figure 12:**
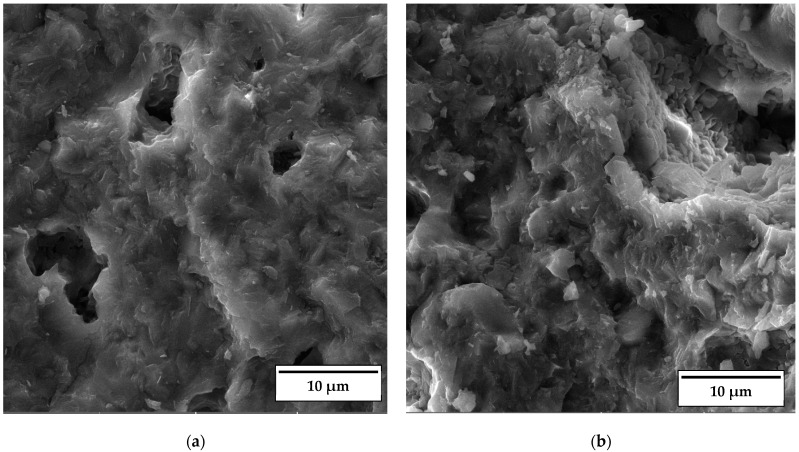
SEM micrograph of fracture surfaces of samples sintered at 1200 °C for 2 h containing (**a**) 0% and (**b**) 30% banana peel.

As documented above, the further increase in the sintering temperature to 1200 °C strongly affected the properties of the anorthite ceramics. The microstructures of the samples containing 0 wt% and 30 wt% banana peel sintered at 1200 °C are presented in Figure 12. The SEM micrographs confirm the results of the porosity measurements given in Figure 6, which show that the samples sintered at 1200 °C possess a considerably lower open porosity than samples sintered at 1100 °C and 1150 °C. Although the comparison of Figure 11b and Figure 12b indicates that the samples containing 30 wt% banana peel sintered at 1200 °C possess large, open pores (>10 µm), the number of these pores is considerably smaller than that in samples sintered at 1150 °C. The decrease in pore number in samples containing 30 wt% banana peel sintered at 1200 °C was accompanied by the largest shrinkage, which reached a value of ~25% (Figure 7). It appears that the porosity introduced by the burnout of banana peel cannot be eliminated easily, even at high temperatures such as 1200 °C. However, the open porosity in samples containing no banana peel that were sintered at 1200 °C was almost completely eliminated (Figure 12a). Insulated pores with a diameter less than 10 µm were observed. This suggests that the driving force for sintering was high enough to eliminate the large pores responsible for premature fracture. Evidently, these samples had the highest compressive strength, 92 MPa, and the highest thermal conductivity at the same time.

## 4. Conclusions

Porous anorthite ceramics, suitable for thermal insulation in buildings, were obtained using waste seashells as a source of CaO, kaolin as a source of Al_2_O_3_ and SiO_2_ and banana peel as a pore former. It was shown that the thermo-mechanical properties of the obtained anorthite ceramics can be effectively controlled by varying the sintering temperature from 1100 to 1200 °C and the amount of banana peel from 0 wt% to 30 wt%. Their bulk density was in the range 1.87–2.62 g/cm^3^, their open porosity was in the range 1.4–45%, their compressive strength was in the range 13–92 MPa and their thermal conductivity was in the range 0.097–3.5 W/mK. Two of the key properties for insulation materials, thermal conductivity and compressive strength, were inversely proportional to the samples’ open porosity. The lowest thermal conductivity of 0.097 W/mK was measured in samples containing 30 wt% banana peel sintered at 1100 °C, which had an open porosity of ~45% and a compressive strength of 13 MPa. On the other hand, the highest thermal conductivity of 3.5 W/mK was measured in the samples without banana peel sintered at 1200 °C, which had an open porosity of 1.4% and a compressive strength of 92 MPa. It can be concluded that samples containing 30 wt% banana peel sintered at 1150 °C are the most appropriate for building insulation. They possess low thermal conductivity and sufficiently high compressive strength.

## Data Availability

Data are contained within the article.
